# A Case Report on the Progressive Hunterian Ligation of an Intracranial Aneurysm by Flow Diversion: The Endovascular Selverstone Clamp

**DOI:** 10.7759/cureus.21218

**Published:** 2022-01-13

**Authors:** Philip Schmalz, Anant Patel, Erik Hauck

**Affiliations:** 1 Department of Neurosurgery, Duke University, Durham, USA

**Keywords:** intracranial aneurysm, flow diversion, endovascular, posterior inferior cerebellar artery

## Abstract

Proximal Hunterian ligation remains a treatment option for select complex brain aneurysms. Progressive occlusion over time (as accomplished with Selverstone clamping) can enable collateral flow to develop while the aneurysm regresses or occludes.

A 50-year-old woman presented with an unruptured 16-mm posterior inferior cerebellar artery (PICA) aneurysm. The aneurysm was located 4 mm distal to the PICA origin. It was bilobed, incorporating the PICA. The PICA inflow and outflow zone orientation prevented direct stent reconstruction. Surgical clipping with bypass was considered. Alternatively, an attempt at proximal ligation of the PICA via flow-diverting stents was offered. After extensive counseling, the patient decided to proceed with endovascular treatment.

Two overlapping pipeline embolization devices (PED) were placed into the vertebral artery, covering the PICA origin. The one-year follow-up angiography demonstrated flow reduction within the aneurysm and the distal PICA. A *de novo* (previously not opacified) accessory PICA collateral had developed, partially taking over the arterial supply of the PICA territory. The newly established accessory PICA originated from the vertebral artery 8 mm distal to the PICA origin. After two years, the aneurysm was fully obliterated, and the true PICA was occluded and functionally replaced by the accessory PICA.

The current case suggests that progressive Hunterian ligation via endovascular flow diversion can be an effective treatment strategy for true PICA aneurysms. However, this strategy should only be considered if no immediate aneurysm occlusion is required or if all alternative methods are associated with substantial risk.

## Introduction

Proximal Hunterian ligation provides a treatment option in cases of select complex brain aneurysms [1]. Progressive occlusion over time (by employing Selverstone clamping) can allow for collateral flow to develop while the aneurysm regresses or occludes [2]. Aneurysms of the posterior inferior cerebellar artery (PICA) are being increasingly treated with endovascular techniques, including flow diversion [1]. However, flow diversion in the posterior circulation is associated with substantial morbidity, largely due to perforator territory infarction [3,4].

We present a patient with accessory PICA formation after undergoing treatment for a PICA aneurysm using the pipeline embolization device (PED). This case involved a true PICA aneurysm; a small segment of angiographically normal PICA was present prior to the aneurysm. Published experience with this type of aneurysm has focused on flow diversion within the PICA. This is technically challenging due to the vessel’s size [5]. These authors report excellent results; however, this case illustrates that aneurysm occlusion may be obtained by flow diversion within the vertebral artery alone.

To our knowledge, this is the first report on the formation of a new PICA-like vessel, recapitulating the territory of the original PICA, after flow diversion. This vessel originated from the vertebral artery, capturing the territory of the native PICA as that vessel regressed. Despite the growing experience with the method, the risk associated with flow diversion in the posterior circulation remains high, with mortality rates over 10% reported in a large series [4]. A better understanding of the hemodynamics and potential tissue response affecting flow diversion in the posterior circulation is required.

## Case presentation

History

A 50-year-old woman presented with acute-onset diplopia and anisocoria with a mild headache. Her medical history was notable for hypertension and osteoarthritis. Social history was negative for the use of tobacco or other drugs. There was no family history of aneurysms.

Examination and hospital course

**Figure 1:**
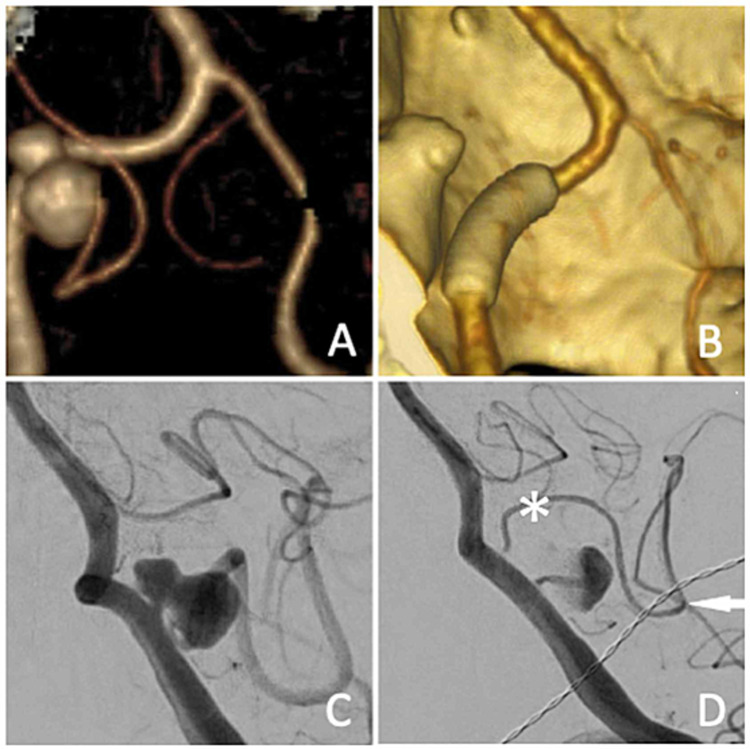
Images obtained during the treatment course A. Three-dimensional reconstruction of CT angiogram obtained on the day of presentation. A bilobed aneurysm is present with discrete PICA inflow and outflow from the larger, inferior lobe. B. The final CT angiogram demonstrates complete occlusion of the aneurysm and the Pipeline stent construct. C. Initial lateral angiogram with injection into the vertebral artery demonstrating the bilobed aneurysm of the “true” PICA. D. Six-month lateral angiogram with injection into the vertebral artery demonstrates sluggish filling of the aneurysm and interval development of an enlarged vertebral artery perforator that recapitulates the territory supplied by the PICA (asterisk) CT: computed tomography; PICA: posterior inferior cerebellar artery

Upon examination, the patient's mental status and speech were normal. There was no objective cranial nerve, sensory, or motor deficit. Laboratory evaluation was unremarkable. She underwent a CT angiogram (CTA) of the head, which was negative for subarachnoid hemorrhage but demonstrated an aneurysm of the left PICA (Figure 1A). Angiography was performed, which showed a fusiform aneurysm of the PICA, arising 4 millimeters after the PICA origin. The PICA continued distally from the aneurysm, arising from the inferior aspect of the second lobe, and followed the usual course of this artery. Contrast-filling the PICA was delayed due to flow resistance caused by the aneurysm (Figure 1C). All complaints of diplopia and mild headaches resolved and she was subsequently discharged. She returned the following month for treatment.

Operation

After informed consent was obtained, the patient was placed under general anesthesia. Transfemoral access to the left vertebral artery was established using an ENVOY® 5 French catheter. Two Pipeline Flex 4 x 14-mm devices (Medtronic, Minneapolis, MN) were delivered through a Marksman microcatheter (Medtronic, Minneapolis, MN) and deployed in the V4 segment of the left vertebral artery, overlapping at the origin of the PICA. This is an off-label application of this device. The use of multiple, overlapping stents achieves better metal coverage of the aneurysm.

Postoperative course

The patient recovered in the neuro ICU. No complications were noted, and she was discharged home on post-procedure day one. A six-month follow-up angiogram was performed, which demonstrated marked flow reduction in the aneurysm and the distal PICA territory beyond (Figure 1D). Blood flow into the distal PICA was delayed, more than what was seen on the pre-treatment angiogram. During the six-month interval, a new vessel originating from the vertebral artery, distal to the native PICA origin but within the stent construct, was seen. This vessel pursued the ordinary course of the PICA and supplied the downstream brainstem and cerebellar territories that would have been supplied by the original vessel (Figure 1D). The final CTA imaging demonstrated complete occlusion of the aneurysm (Figure 1B).

## Discussion

The development of the central nervous system (CNS) vasculature has been described as a longitudinal neural system with transversely oriented segmental vessels connecting at regular intervals. This system is refined throughout the development, with certain vessels expanding to supply greater territory, while others regress [6]. The end result is a complementary relationship between adjacent transverse vessels. These vessels have variable downstream anastomoses and a complex relationship with both large branches and smaller perforators, any of which may supply brainstem territories [6].

This case demonstrates the use of flow diversion within the vertebral artery for the treatment of a true PICA aneurysm. The mechanism of aneurysm occlusion can be viewed as a progressive Hunterian ligation. This case also highlights the utility of the complex complementary relationships between the PICA, vertebral artery, and their associated brainstem perforators. Endovascular treatment of PICA aneurysms has been extensively described in the literature, with distal lesions often treated with vessel sacrifice and proximal aneurysms usually managed with coil embolization [7,8]. Flow diversion is an attractive option for endovascular treatment of these aneurysms to preserve both the PICA and the perforators to the brainstem that arise from the PICA and vertebral arteries. Experience with flow diverters for the treatment of true PICA aneurysms has focused on the placement of the flow diverter within the PICA [5]. While this has been successful, flow diversion within the PICA is more challenging. Placement in the vertebral artery, in this case, was both technically straightforward and effective.

The fate of covered branch arteries has been extensively studied for the ophthalmic and vertebral arteries [9,10]. These studies indicate that branch occlusion is more likely to occur when there is a greater collateral supply to the territory supplied by the covered vessel. Placement of multiple devices, as in our case, also increases the risk of branch occlusion but also increases the likelihood of successful aneurysm obliteration. Careful attention to anatomy and collaterals is crucial when making decisions on placing multiple devices.

In this case, the recruitment of a large perforating artery to supply the territory of the existing PICA facilitated aneurysm occlusion. A thorough understanding of angiographic features, which may predict ischemia or facilitate aneurysm occlusion, will improve patient selection for flow diversion treatment.

## Conclusions

Endovascular treatment of a PICA aneurysm by flow diversion, an endovascular Hunterian ligation, within the vertebral artery can result in the remodeling of the PICA and aneurysm resolution. Perforating arteries arising from the vertebral artery may develop to supply the territory previously supplied by the PICA. This case highlights the efficacy of flow diversion within the vertebral artery for PICA aneurysms and the complementary relationships that must be understood to perform flow diversion in the posterior circulation. Neurointerventionists treating similar aneurysms should consider collateral flow and vessel recruitment to improve safety and efficacy.
